# Anti-caries effect of a novel elastic silicone appliance material incorporating sodium fluoride

**DOI:** 10.3389/fmicb.2024.1517188

**Published:** 2025-01-06

**Authors:** Shuxing Yu, Lingyu Zhang, Qizhao Ma, Jing Zhou, Yaqi Liu, Jing Zou, Qiong Zhang

**Affiliations:** ^1^State Key Laboratory of Oral Diseases & National Center for Stomatology & National Clinical Research Center for Oral Diseases, Department of Pediatric Dentistry, West China Hospital of Stomatology, Sichuan University, Chengdu, Sichuan, China; ^2^Department of Stomatology, Affiliated Hospital of Shandong University of Traditional Chinese Medicine, Jinan, China; ^3^State Key Laboratory of Oral Diseases & National Center for Stomatology & National Clinical Research Center for Oral Diseases, Department of Pediatric Dentistry, Department of Jinjiang Outpatient, West China Hospital of Stomatology, Sichuan University, Chengdu, Sichuan, China

**Keywords:** elastic silicone appliance material, sodium fluoride, *Streptococcus mutans*, mechanical properties, cytotoxicity, enamel hardness

## Abstract

**Introduction:**

This study developed an elastic silicone appliance material incorporating sodium fluoride (NaF) and evaluated its mechanical properties, biocompatibility, antibacterial effects, and remineralization potential.

**Methods:**

Silicone components A and B were combined with varying concentrations of NaF (0.5, 1, 1.5, 2, and 2.5%), thoroughly mixed, and transferred into molds. After drying and curing, the resulting orthodontic appliance was retrieved from the mold and underwent finishing processes, followed by the assessment of its mechanical properties, cytotoxicity, and antibacterial impact. Additionally, the impact of this novel silicone appliance material on salivary biofilm’s activity and acid production was evaluated in samples from children with severe early childhood caries (S-ECC). The hardness of demineralized and remineralized bovine enamel was measured.

**Results:**

Incorporating NaF (0.5, 1, and 1.5%) resulted in no cytotoxic effects, with cell viability >85%. The fluoride release rate initially increased over 14 days, followed by a gradual decline, maintaining a steady release for approximately 28 days. Incorporating 1.5% NaF preserved the mechanical properties and exhibited specific antibacterial properties that inhibited the growth, biofilm formation, and acid production activity of *Streptococcus mutans* (*S. mutans*) and saliva biofilms from S-ECC children. Furthermore, all concentrations of the samples helped improve enamel hardness loss.

**Discussion:**

The novel silicone appliance material incorporating NaF exhibited antibacterial, fluoride releasing, and enamel remineralization properties while maintaining its physical and chemical integrity without cytotoxic effects.

## 1 Introduction

In recent years, an increasing number of patients have opted for esthetically pleasing and comfortable invisible orthodontic appliances. Compared with fixed appliances, these aligners’ transparency and low rigidity reduce discomfort, enhance quality of life, and improve esthetics during orthodontic treatment. However, it is important not to overlook orthodontic appliances’ direct or indirect impacts on the oral microbiome and periodontal tissues (Rossini et al., 2015). Recent research indicated that removable orthodontic appliances do not significantly alter the oral microbiota structure; instead, they offer distinct advantages over fixed orthotic appliances regarding the plaque index, gingival health, and white spot lesion (WSL) prevalence (Rouzi et al., 2023). Nevertheless, grooves, ridges, microcracks, and surface wear on clear orthotic appliances create a favorable environment for bacterial adhesion and plaque biofilm formation, facilitating the onset and progression of common oral microbial diseases (Metin-Gürsoy et al., 2017; Abou Neel et al., 2016; Xu et al., 2020). Furthermore, research has indicated no substantial disparity in the elevated levels of the primary cariogenic bacteria *S. mutans* between individuals using invisible orthodontic appliances and those wearing fixed appliances (Sifakakis et al., 2018; Mummolo et al., 2020). Regardless of the appliance type, orthodontic patients are more likely to develop WSLs compared to non-appliance users (Bisht et al., 2022). Enamel demineralization is a part of the caries process, initially presented clinically as WSLs (Sardana et al., 2023). During this process, the activity of bacterial plaque and the release of acid from carbohydrate metabolism increase (Upadhyay et al., 2013), leading to cavity formation (Francci et al., 1999).

*Streptococcus mutans* exhibits significant acid production, resistance, and extracellular polysaccharide (EPS) adhesion to the tooth surface. It can also adapt to the oral environment to regulate the composition and pathogenicity of the mature biofilm community (Lamont et al., 2018; Kolenbrander, 2000; Loesche, 1986; Lemos et al., 2019). *S. mutans* metabolizes sugars to produce acid, demineralizing hydroxyapatite (HA) crystals in hard tissues such as enamel, dentin, cementum, and bone. The process by which these mineral ions are restored is referred to as remineralization (Finke et al., 2000). Both processes occur simultaneously on the tooth surface, where large amounts of mineral ions in HA can be lost without damaging its integrity (Hart and Hart, 2007).

Fluoride ions can reduce enamel’s solubility by forming fluorapatite (FAP) and are beneficial for enamel remineralization to protect demineralized enamel (Ten Cate, 1990; Featherstone et al., 1990). In addition, they inhibit bacterial growth and metabolism by inhibiting the activity of enzymes such as enolase and ATPase (Oh et al., 2017; Ten Cate, 2004). When the environmental pH decreases, the protons and fluoride ions can diffuse into bacterial cells and emerge in the cytoplasm as hydrogen fluoride, inhibiting the growth of *S. mutans* and its cariogenic virulence (Marquis et al., 2003; Liao et al., 2017). Partially demineralized HA may be restored to its original hardness if exposed to an oral environment conducive to remineralization, reducing the risk of deeper cavities (Selwitz et al., 2007). Fluoride compounds, such as sodium fluoride (NaF), have become an active, popular, and effective way to prevent dental caries (Nagireddy et al., 2019; Jones and Worthington, 2000). Orthodontic patients usually receive oral hygiene instructions before orthodontic treatment begins. Orthodontic patients with a high caries risk are recommended to regularly use fluoride varnish, which requires additional time and expense; however, younger patients have difficulty following this guidance (Chambers et al., 2013; Featherstone, 1999). Therefore, it is absolutely necessary to prevent WSL and cavities by other means.

Current research has indicated that fluoride-coated clear aligner plastic (FCAP) effectively prevents the development of WSL and progressive caries (Yan et al., 2023). However, the coating on the surface will inevitably be worn when the removable appliance is removed for cleaning. Hence, this method represents a novel and efficacious approach to developing appliances with sustained fluoride release. This study investigated the effects of a novel silicone appliance material incorporating NaF on the antibacterial activity and the possibility of inhibiting WSL and even preventing the formation of cavities.

## 2 Materials and methods

### 2.1 Mechanical characteristics and biological properties of the specimen

#### 2.1.1 Specimen preparation

Silicone components A and B were combined with varying concentrations of NaF (0.5, 1, 1.5, 2, and 2.5%) and thoroughly mixed. The mixture was then transferred into molds, dried, and set. The fabricated orthodontic appliances were retrieved from the molds and subjected to finishing procedures (Supplementary Figure S1). Circular silicone samples measuring 10 mm in diameter and 2 mm in thickness were prepared (Beijing Appliance Health Technology Co., Ltd). The samples were sterilized using ethylene oxide at 37°C.

#### 2.1.2 Mechanical properties of the specimen

Each group’s Shore hardness was measured using a Shore durometer (Kim et al., 2014). The tensile strength, tear strength, flexural elastic modulus, and tensile elastic modulus were evaluated using rectangular specimens, whereas the compressive elastic modulus was assessed using circular specimens (Criterion C45, MTS, US). All the measurements were recorded.

#### 2.1.3 Cytotoxicity test of the specimen

(3-(4,5-dimethylthiazol-2-yl)-2,5-diphenyltetrazolium bromide) and CCK8 (cell counting kit-8) were used as described previously (Lü et al., 2009; Zhang et al., 2019).

The samples were sterilized using ethylene oxide‌ to prepare the leachate. Subsequently, they were immersed in a solution containing 2 mL of Minimum Essential Medium (MEM) and Dulbecco’s Modified Eagle Medium (DMEM), both sourced from Gibco, USA and supplemented with 2% fetal bovine serum, along with antibiotics (100 IU/mL penicillin and 100 IU/mL streptomycin). The immersion process was conducted at 37°C for 24 h to generate the leachate required for subsequent MTT and CCK8 cell toxicity assays (Zhang et al., 2013).

##### 2.1.3.1 Cytotoxicity was assessed using the MTT assay

###### 2.1.3.1.1 Cell seeding

Murine fibroblast L-929 cells were cultured at 37°C under 5% CO_2_. Cell suspensions were prepared by trypsin treatment, seeded into 96-well plates at 10,000 cells/well density, and incubated for 24 h (Malkoc et al., 2010).

###### 2.1.3.1.2 Adding test substance

After incubation, the culture medium was discarded, and 100 μL of sample leachate was added to each well. The plates were subsequently incubated for an additional 24 h, and a blank control group containing only culture medium was included. The plates were incubated for another 24 h.

###### 2.1.3.1.3 MTT (ST316, Beyotime, Shanghai, China) assay

For incubation with the MTT solution, 50 mL of MTT solution (1 mg/mL, ST316, Beyotime, Shanghai, China) was added to each well, followed by a 2-h incubation.

###### 2.1.3.1.4 Dissolution of formazan crystals

The supernatant was carefully removed, and 1 mL of dimethyl sulfoxide (DMSO, ST038, Beyotime, Shanghai, China) was added to dissolve Formazan crystals. The plates were gently oscillated for 20 min.

###### 2.1.3.1.5 Measurement of absorbance

Absorbance was measured at 570 nm using a microplate reader to reflect the number of viable cells. Cell viability was calculated using the formula: $\left(\frac{\mathrm{experimental group}-\mathrm{blank group}}{\mathrm{control group}-\mathrm{blank group}}\right)\times 100\%$ (experimental group: cells treated with sample leachate; control group: cells treated only with culture medium, without any leachate treatment; blank group: only culture medium, with no cells present). This method was used to evaluate the cytotoxic effects of the test substances.

##### 2.1.3.2 Cytotoxicity was assessed using the CCK8 assay

###### 2.1.3.2.1 Cell seeding

Human gingival epithelial (Hge), and human oral keratinocyte (Hok) cells were cultured at 37°C under 5% CO_2_. Cell suspensions were prepared by trypsin treatment, seeded into 96-well plates at a density of 10,000 cells/well, and incubated for 24 h (Zhang et al., 2019).

###### 2.1.3.2.2 CCK8 (K1018, APE×BIO, UAS) assay

10 μL of CCK8 solution (according to the instructions) was added to each well, and the Hge and Hok cells were then incubated for an additional 1 h.

###### 2.1.3.2.3 Measurement of absorbance

Absorbance was measured at 450 nm using a microplate reader to determine the number of viable cells. The cell viability was calculated using the formula: $\left(\frac{\mathrm{experimental group}-\mathrm{blank group}}{\mathrm{control group}-\mathrm{blank group}}\right)\times 100\%$ (experimental group: cells treated with sample leachate; control group: cells treated only with culture medium, without any leachate treatment; blank group: only culture medium, with no cells present). This method was used to evaluate the cytotoxic effects of the test substances.

#### 2.1.4 Fluoride release capacity of the specimen

Each group consisted of four samples with 0, 0.5, 1%, or 1.5% NaF. Each sample was immersed in 500 mL of distilled water (pH = 7.0) at room temperature for 8 h daily to simulate the release of fluoride ions. Following immersion, the samples were dried for 16 h to replicate the daily wearing schedule of orthodontic appliances in children. Total Ionic Strength Adjustment Buffer II (TISAB II, Phygene, China) was added to the leaching solution at a 1:1 ratio to determine fluoride ion concentration. Measurements were made daily for 8 weeks using a fluoride ion-selective electrode (7,102, Fangzhou Science and Technology, Beijing, China).

### 2.2 Anti-biofilm and anti-caries properties of the specimen

#### 2.2.1 Bacteria, media, and growth conditions

*Streptococcus mutans* UA159 (ATCC 700610) was cultured in brain heart infusion (BHI) broth (Difco, Sparks, MD, USA) under anaerobic conditions (85% N_2_, 10% H_2_, 5% CO_2_) at 37°C. Biofilms were developed using BHI supplemented with 1% sucrose (BHIS).

#### 2.2.2 S-ECC saliva sampling and saliva-derived biofilm formation

An *in vitro* dental plaque microecological model was developed using human saliva, producing a biofilm that closely mimicked oral plaque biofilms and contained most microbial species found in dental plaque (Exterkate et al., 2010). Studies have demonstrated distinct differences in the microbiota between caries-free individuals and those with a high risk for caries (Abou Neel et al., 2016; Wang et al., 2006; Schwendicke et al., 2016). To represent oral microbial species associated with clinical caries risk, saliva was collected from children with S-ECC. Saliva collection was approved by the Ethics Committee of West China Hospital of Stomatology, Sichuan University (WCHSIRB-D-2024-083). After obtaining informed consent from guardians, non-stimulated salivary samples were collected from the participants and transferred to the laboratory on ice within 2 h. Samples from multiple participants were pooled, mixed with an equal volume of 50% glycerol, and stored at −80°C for future use (Cheng et al., 2012). For biofilm formation, 2 mL of SHI medium was inoculated with the saliva storage solution under anaerobic conditions (85% N_2_, 10% H_2_, 5% CO_2_) and cultured at 37°C for 24 h (Tian et al., 2010). Circular specimens were placed in 24-well plates, immersed in 2 mL of SHI medium, seeded with saliva-glycerol stock at a 1:30 ratio, and incubated anaerobically (90% N_2_, 5% CO_2_, 5% H_2_) at 37°C for 24 h.

#### 2.2.3 Antimicrobial activity against *Streptococcus mutans*

*Streptococcus mutans* was cultured overnight, and the bacterial suspension was inoculated into fresh BHI medium at a ratio of 1:100. The culture was grown to the mid-logarithmic phase (OD_600_ = 0.5) and diluted 1:100 (v/v) in 2 mL of BHI. The diluted suspension was then cultured with specimens incorporating 0.5, 1, and 1.5% NaF for 8 h. The supernatant’s optical density (OD600) was measured using a microplate reader (BioTek, USA).

#### 2.2.4 Biofilm formation assay

*Streptococcus mutans* was cultured overnight, as described previously, and the bacterial suspension was inoculated into fresh BHI medium at a ratio of 1:100 and cultured to the exponential phase (OD_600_ = 0.5). The culture was then diluted 1:100 (v/v) in 2 mL of 1% BHIS and incubated with specimens containing 0.5, 1, and 1.5% NaF for 4, 8, and 12 h. The supernatant was discarded after incubation, and each well was rinsed with phosphate-buffered saline (PBS) three times. The biofilms were fixed with 4% polyformaldehyde and dried at 37°C, followed by biofilm biomass quantification using crystal violet staining (Pan et al., 2021). The wells were stained with 0.01% crystal violet for 15 min, rinsed with PBS, and the dye was dissolved in 33% ice acetic acid. The antibiofilm’s ability was assessed by measuring the optical density at 575 nm using a microplate reader (BioTek, USA).

#### 2.2.5 Observation of biofilm structure and morphology

##### 2.2.5.1 Scanning electron microscope (SEM) experiment

*Streptococcus mutans* was cultured overnight. The bacterial suspension was inoculated into fresh BHI medium at a ratio of 1:100 and cultured to the logarithmic growth phase (OD_600_ = 0.5). Regarding biofilm formation, *S. mutans* was cultured with specimens containing 0.5, 1, and 1.5% NaF on sterile cell slides placed in 12-well plates using BHIS medium for 24 h. The medium was discarded after incubation, and the cells were gently washed with PBS three times. The biofilms were fixed in 2.5% glutaraldehyde for at least 3 h, followed by dehydration with a graded ethanol series (30, 40, 50, 60, 70, 90, and 100%). The slides were freeze-dried overnight, sputter-coated with gold, and observed under a scanning electron microscope (SEM; Quanta 200, FEI, Hillsboro, Oregon, USA).

##### 2.2.5.2 Structural imaging of biofilms using a confocal laser scanning microscope (CLSM)

*Streptococcus mutans* cultures were grown overnight, and the bacterial suspension was subsequently inoculated into fresh BHI medium at a dilution ratio of 1:10. After reaching the logarithmic phase (OD_600_ = 0.5), the culture was transferred to glass-bottomed cell dishes containing specimens with 0, 0.5, 1, and 1.5% NaF concentrations. To culture *S. mutans* biofilm, Alexa Fluor 647 glucan conjugate (119 Invitrogen, Carlsbad, CA, USA) was mixed with BHIS to a final concentration of 1 μM before culturing. After incubation, the cover plate was rinsed with normal saline (NS) twice to remove floating and loosely bound cells. Subsequently, *S. mutans* was stained with 2.5-μM SYTO 9 (Molecular Probes, Invitrogen, Carlsbad, CA, USA) for 15 min, followed by two washes with NS (Chen et al., 2021). An antifluorescence quencher (S2110, Solarbio, China) was then added. Images were captured using a Nikon CLSM (Nikon, N-SIM) with a 60× oil-immersed objective lens. Image acquisition gates were set at 495–515 nm for SYTO 9 and 655–690 nm for Alexa Fluor 647. Each biofilm was scanned at three randomly selected locations.

#### 2.2.6 MTT metabolic assay

Saliva-derived biofilms were cultured as described previously, the medium was discarded, and the cells were washed with PBS to remove planktonic cells. The metabolic activity of salivary S-ECC biofilms was evaluated using the MTT assay (Hashemi et al., 2019). Briefly, 200 μL of fresh broth containing 0.5-mg/mL MTT was added to each well, and the plate was incubated in the dark at 37°C for 4 h. The solution was discarded after incubation, and 100 μL of dimethyl sulfoxide (DMSO) was added to each well, followed by gentle shaking of the plate for 10 min to dissolve the formazan crystals. The absorbance was measured at 570 nm using a microplate reader.

#### 2.2.7 Glycolytic pH drop

As mentioned earlier, the acid production capacity of *S. mutans* through glycolysis has undergone some modifications (Belli and Marquis, 1991). In brief, specimens (incorporating 0.5, 1, and 1.5% NaF) were immersed in 2 mL of 0.5-mM potassium phosphate buffer (PPB), which contained 37.5-mM KCl and 1.25-mM MgCl_2_ (pH = 7), and removed after 8 h to obtain specimen leachate. 2 mL of *S. mutans* was harvested at OD_600_ = 0.5, centrifuged, and resuspended in the specimens. Glucose (1%) was added to the PPB solution, and the pH of the solution was monitored after *S. mutans* glycolysis using a Benchtop Meter (Thermo Scientific, Waltham, MA).

#### 2.2.8 Biofilm acid production experiment

The S-ECC salivary biofilm samples were rinsed with PBS after 24 h of culture and transferred to a new 24-well plate. 2 mL buffered peptone water (BPW) containing 1% sucrose was added to each well at 37°C, and an anaerobic culture was performed for 4 h. The lactic acid concentration in each well was measured using a lactic acid test kit (Nanjing Jiancheng Bioengineering Institute, China) after the samples were removed (Chen et al., 2022). One-way ANOVA and Tukey multiple comparison tests were used to analyze the data in each group at a significance level of 0.05.

#### 2.2.9 Enamel hardness test

Bovine teeth free of lesions and cracks were sectioned into rectangular enamel blocks (4 × 4 × 2 mm), which were treated for 24 h in a demineralizing solution containing 75-mM acetic acid (pH = 4), 8.7-mM CaCl₂, 8.7-mM KH₂PO₄, and 0.05-ppm NaF, followed by ultraviolet disinfection for 4 h (Gao et al., 2020). Demineralized enamel blocks were co-incubated with samples of varying NaF concentrations in 2 mL of deionized water for 24 h. Enamel hardness was determined using a Shimadzu HMV-2000 hardness tester (Kyoto, Japan) before and after demineralization and after incubation with the samples (Huang et al., 2022).

In a separate assay, demineralized enamel blocks were further demineralized in an acidic SHI medium for 24 h, after which the medium was replaced. The blocks were then exposed to samples containing 0.5, 1%, or 1.5% NaF and cultured for an additional 24 h before hardness testing.

Unmineralized enamel blocks were also incubated with S-ECC salivary biofilm in 2 mL of SHI medium containing samples of varying NaF concentrations for 24 h before testing.

Enamel blocks were first demineralized using the standard protocol for sequential demineralization and remineralization. Then, the samples were immersed daily in 2 mL of a demineralizing solution (3.0-mM CaCl_2_, 1.8-mM KH_2_PO_4_, 0.1-M lactic acid, and 1% carboxymethyl cellulose at pH = 4.0) for 1 h, followed by incubation in 2 mL of a recirculating remineralizing solution (1.2-mM CaCl_2_, 0.72-mM KH_2_PO_4_, 0.05-ppm NaF, and 50-mM HEPES buffer at pH = 7.0) for 8 h. This process was repeated five times before measuring enamel hardness.

### 2.3 Statistical analysis

Statistical analysis was conducted using SPSS 22.0 (SPSS, Inc., Chicago, IL, USA). One-way ANOVA was used to assess the significant impact of a variable. The Tukey multiple comparison tests were used to compare the mean of each group at *p* < 0.05.

## 3 Results

### 3.1 Mechanical properties of the novel silicone appliance material

**Figure 1:**
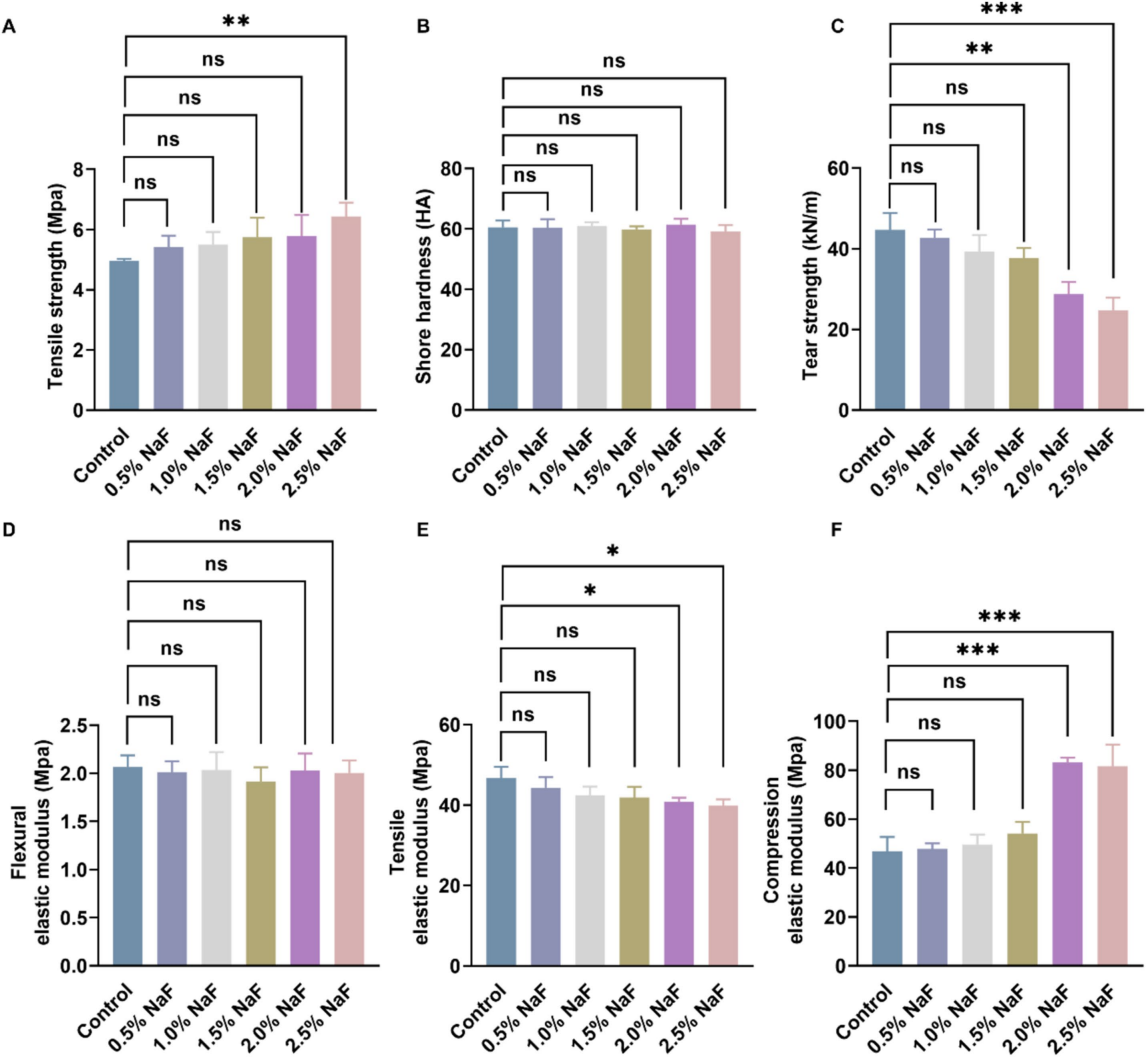
Mechanical properties of silicone appliance material. **(A)** Tensile strength. **(B)** Shore hardness. **(C)** Tear strength. **(D)** Flexural elastic modulus. **(E)** Tensile elastic modulus. **(F)** Compression elastic modulus. There was no significant difference among the four groups incorporating 0, 0.5, 1, and 1.5% NaF. Each experiment was repeated at least three times. The results are presented as mean ± SD (*p < 0.05, **p < 0.01 or ***p < 0.001).

The mechanical properties of the novel silicone appliance were evaluated by testing Shore hardness, tear strength, tensile strength, flexural elastic modulus, tensile elastic modulus, and compression elastic modulus. The results, shown in Figure 1, indicate that incorporating NaF at concentrations of 0.5, 1, and 1.5% did not significantly affect the mechanical properties of the appliance (*p* > 0.05). However, 2% NaF was associated with significant changes in tensile strength, tear strength, tensile elastic modulus, and compression elastic modulus.

### 3.2 Cytotoxicity test of the silicone appliance material

**Figure 2:**
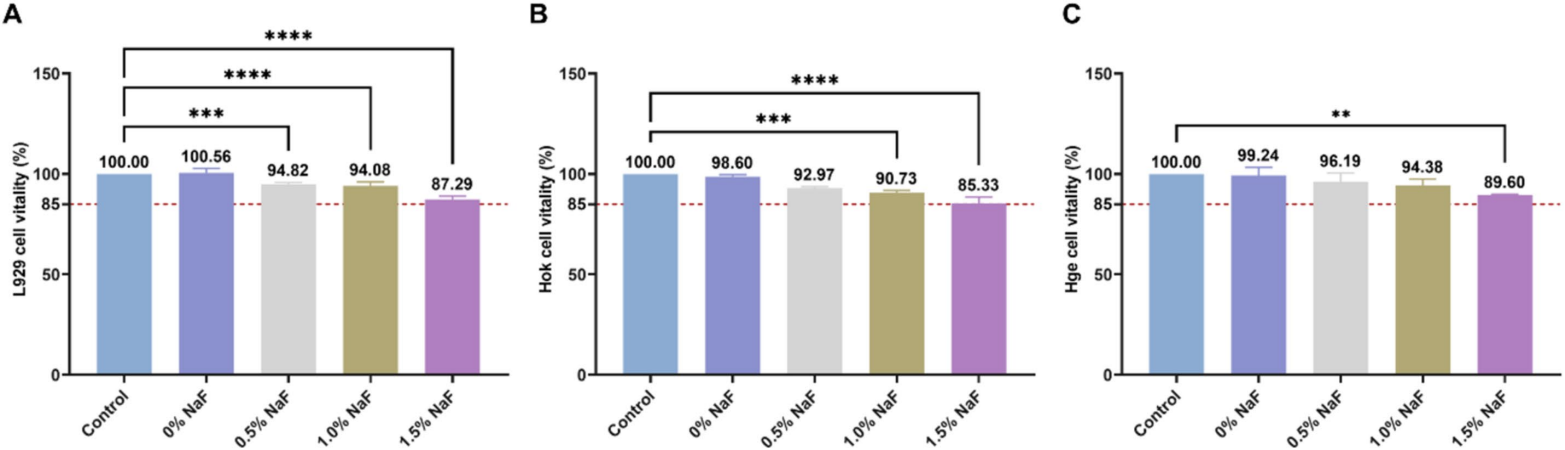
Cytotoxicity of the extracts from each group. **(A)** The effect of silicone appliance material with different concentrations of NaF on L929 cell viability was observed through the MTT assay. **(B)** The effect of silicone appliance material with different concentrations of NaF on Hok cell viability was observed through the CCK8 assay. **(C)** The effect of silica gel medical device materials with different concentrations of NaF on Hge cell viability was observed through the CCK8 assay. According to ISO 10993-5, if cell viability exceeds 80% of the control group, it is considered acceptable. In accordance with ISO 10993-5, cell viability is considered acceptable if it exceeds 80% of the control group (**p < 0.01, ***p < 0.001 or ****p < 0.0001).

The cytotoxicity of the silicone appliance material was assessed using murine L-929 fibroblast, human gingival epithelial, and human oral keratinocyte cells. Cell viability with NaF concentrations of 0, 0.5, 1, and 1.5% was approximately >85%, as shown in Figure 2.

### 3.3 Fluoride release properties of the novel silicone appliance material

**Figure 3:**
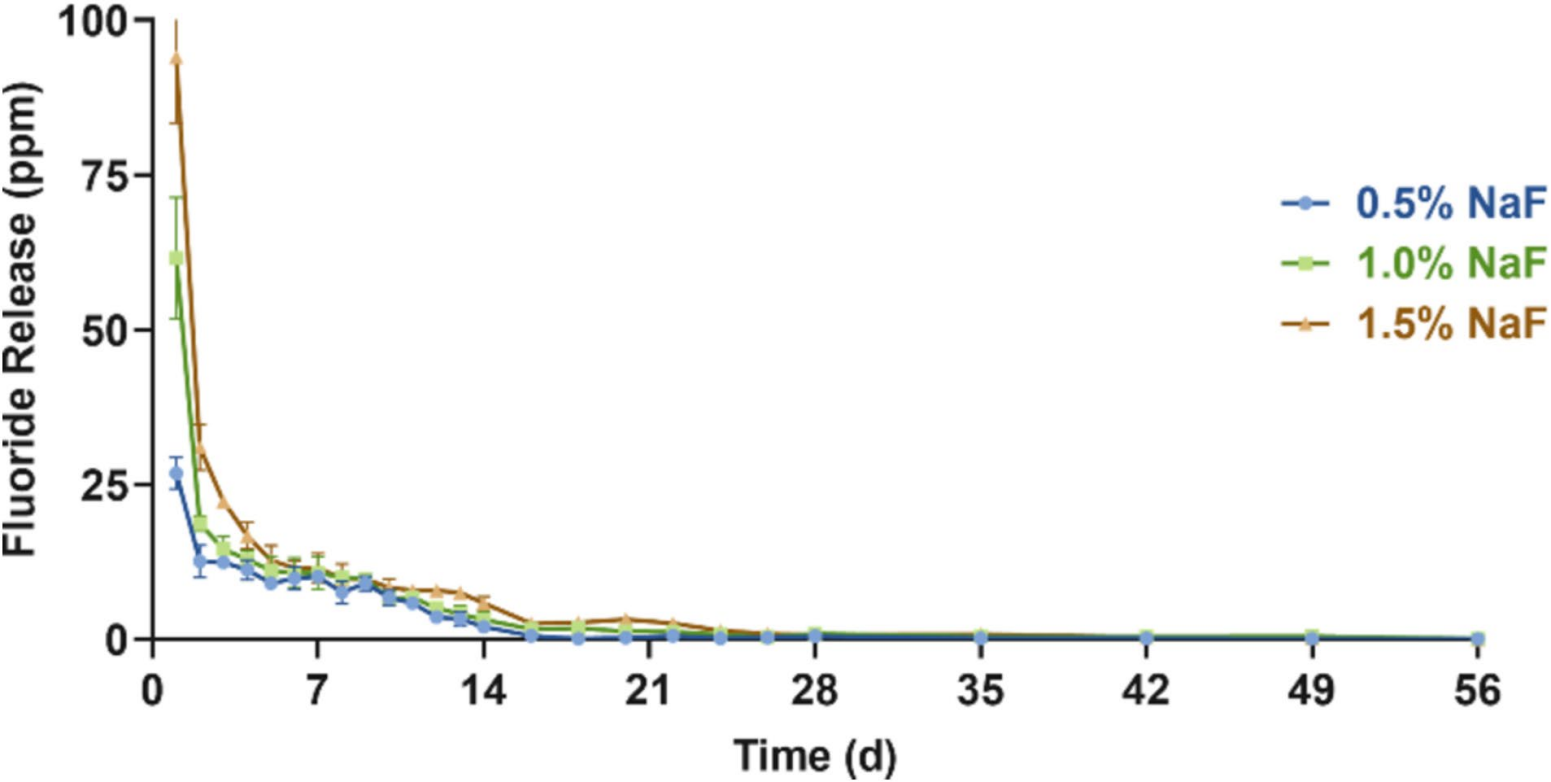
Fluoride release assessment between the three groups incorporating 0.5, 1, and 1.5% NaF. The results are presented as mean ± SD.

Figure 3 presents the fluoride release profiles of each group over 56 days. All the groups exhibited a similar trend, with the highest fluoride release on day 1, followed by a sharp decline. From day 3 to approximately day 28, a relatively stable, low-level fluoride release was maintained. Additionally, higher NaF concentrations in the circular samples corresponded to higher fluoride release, with no statistically significant differences between the groups (*p* > 0.05).

### 3.4 Impact of the novel silicone appliance material on the growth and cariogenic potential of *Streptococcus mutans*

**Figure 4:**
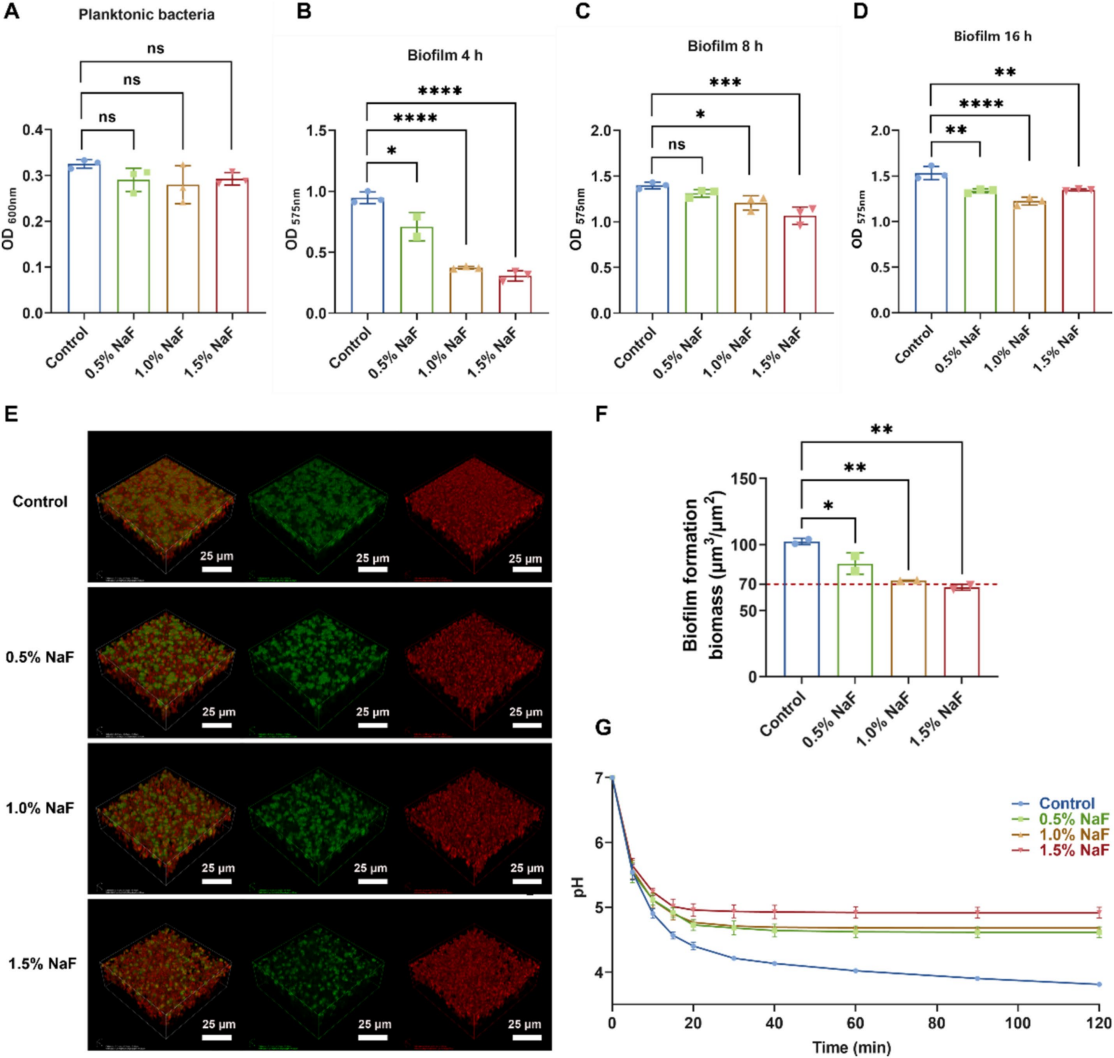
Effect of silicone appliance material incorporating 0.5, 1, and 1.5% NaF on the growth, biofilm formation, and acid production of *S. mutans*. **(A)** Growth characteristics of *S. mutans* in co-culture system with silicone appliance material. **(B–D)** The biofilm biomass of *S. mutans* was determined using a crystal violet staining assay cultured with a silicone appliance containing NaF for 4, 8, and 12 h, respectively. **(E,F)** 24-h biofilms examined by confocal microscopy. Three-dimensional construction was performed using Imaris 7.0.0 (Bitplane). Bacteria are shown in green (SYTO 9), and water-insoluble exopolysaccharides are in red (Alexa Flour 647). Representative images are taken from at least three randomly selected fields from each sample. **(G)** The pH drop curves of *S. mutans* were obtained over 2 h. Each experiment was repeated at least three times. The results are presented as mean ± SD (*p < 0.05, **p < 0.01, ***p < 0.001 or ****p < 0.0001).

Incorporating NaF into the silicone appliance material inhibited the survival and cariogenic potential of *S. mutans*. Growth curve experiments revealed no significant suppression of *S. mutans* survival at the tested NaF concentrations (Figure 4A, *p* > 0.05). However, crystal violet staining revealed significant suppression of biofilm formation at NaF concentrations of 0.5, 1, and 1.5% during the rapid growth and plateau phases (Figures 4B–D, *p* < 0.05).

An increase in the concentration of NaF in the silicone appliance material led to a gradual decrease in *S. mutans* UA159 counts, represented by green. The amount of water-insoluble extracellular polysaccharides, depicted in red, decreased slightly. The overall biofilm mass showed a clear and consistent decline as the concentration of NaF within the silicone appliance samples increased (Figures 4E,F). Scanning electron microscopy (SEM) images (Supplementary Figure S2) revealed the morphology and structure of *S. mutans* biofilms. The control group exhibited a complex mesh structure of extracellular polymeric substances (EPS) and bacterial colonies, with EPS bridging microcolonies to form aggregates. In contrast, with increasing NaF concentrations, EPS became less prominent, and the biofilm structure appeared disrupted, indicating that the silicone appliance material inhibited biofilm formation, with higher NaF concentrations exhibiting stronger effects due to fluoride release.

A pH drop experiment was conducted to assess the impact of NaF on acid production by *S. mutans*. As shown in Figure 4G, NaF incorporation significantly reduced the acid production capacity of *S. mutans*, with a greater reduction at higher NaF concentrations. These findings suggest that the NaF-containing silicone appliance material effectively impairs the growth, biofilm formation, and acid-producing activity of *S. mutans*.

### 3.5 Impact of novel silicone appliance material on MTT metabolism and acid production of saliva-derived biofilms

**Figure 5:**
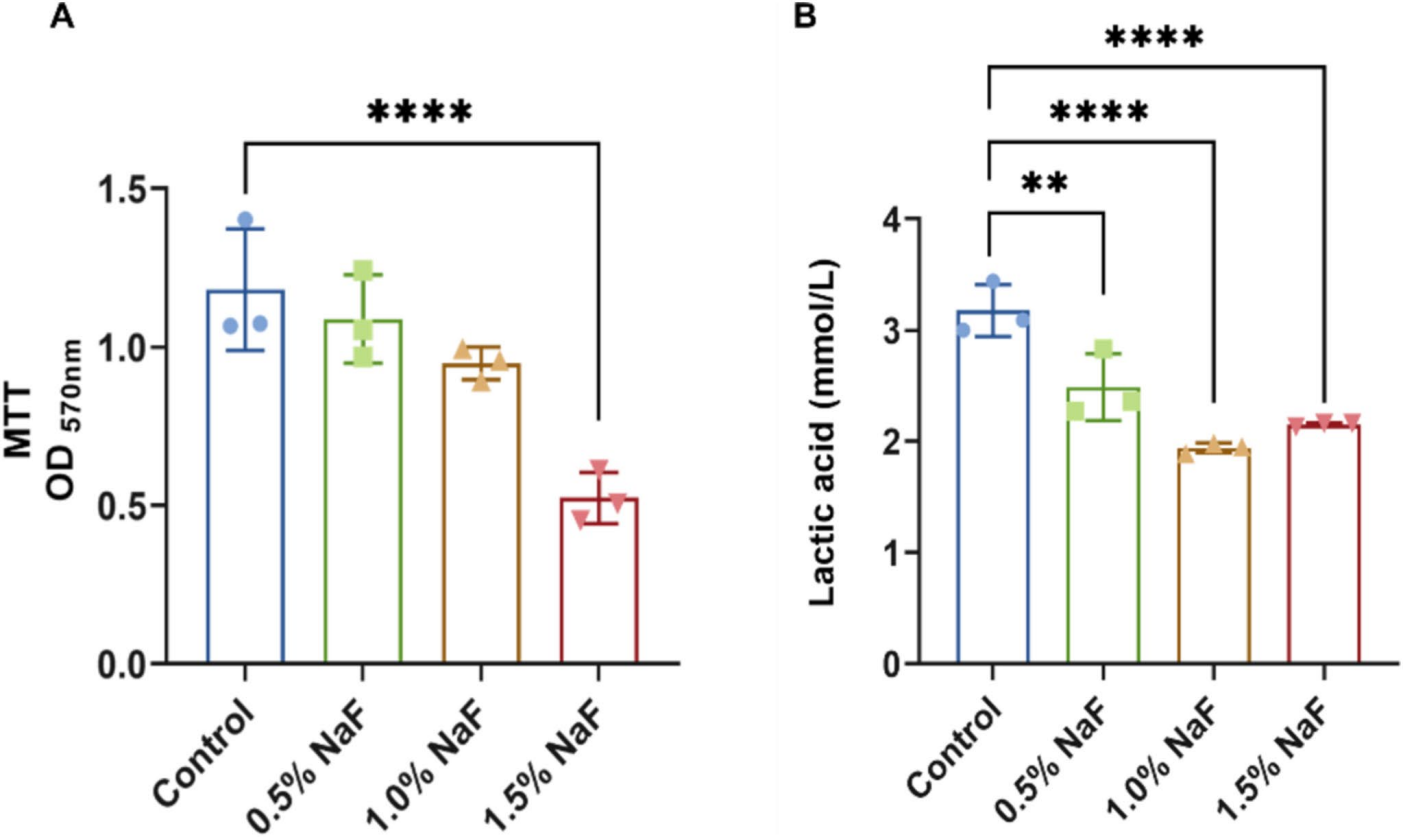
Antibacterial effects of silicone appliance material on saliva-derived biofilm. **(A)** The biofilm metabolism evaluation. **(B)** The production of lactic acid at the biofilm sites. Each experiment was repeated at least three times. The results are presented as mean ± SD (**p < 0.01 or ****p < 0.0001).

Plaque biofilms maintain a dynamic balance under normal conditions. However, when this balance is disrupted, bacteria such as *S. mutans* and *Lactobacillus* proliferate, metabolizing dietary carbohydrates into acids, demineralizing hard dental tissues, and forming cavities. Thus, inhibiting the acid production, metabolism, and growth activity of dental plaque biofilms is critical for caries prevention. MTT metabolic activity was assessed after 24 h, with absorbance values shown in Figure 5A. No significant differences were observed between the 0.5 and 1% NaF sample groups and the control group. However, samples containing 1.5% NaF exhibited significantly lower absorbance compared to the control, indicating reduced metabolic activity. Higher NaF concentrations were associated with lower absorbance values, suggesting a concentration-dependent inhibition of biofilm metabolism. The acidogenic capacity of S-ECC saliva-derived biofilms was also evaluated. The initial pH of the control and silicone appliance material extracts was recorded at approximately 6.8. As shown in Figure 5B, all three NaF concentrations significantly inhibited acid production compared to the control group (*p* < 0.05).

### 3.6 Influence of novel silicone appliance material on the demineralization and remineralization properties of dental enamel

**Figure 6:**
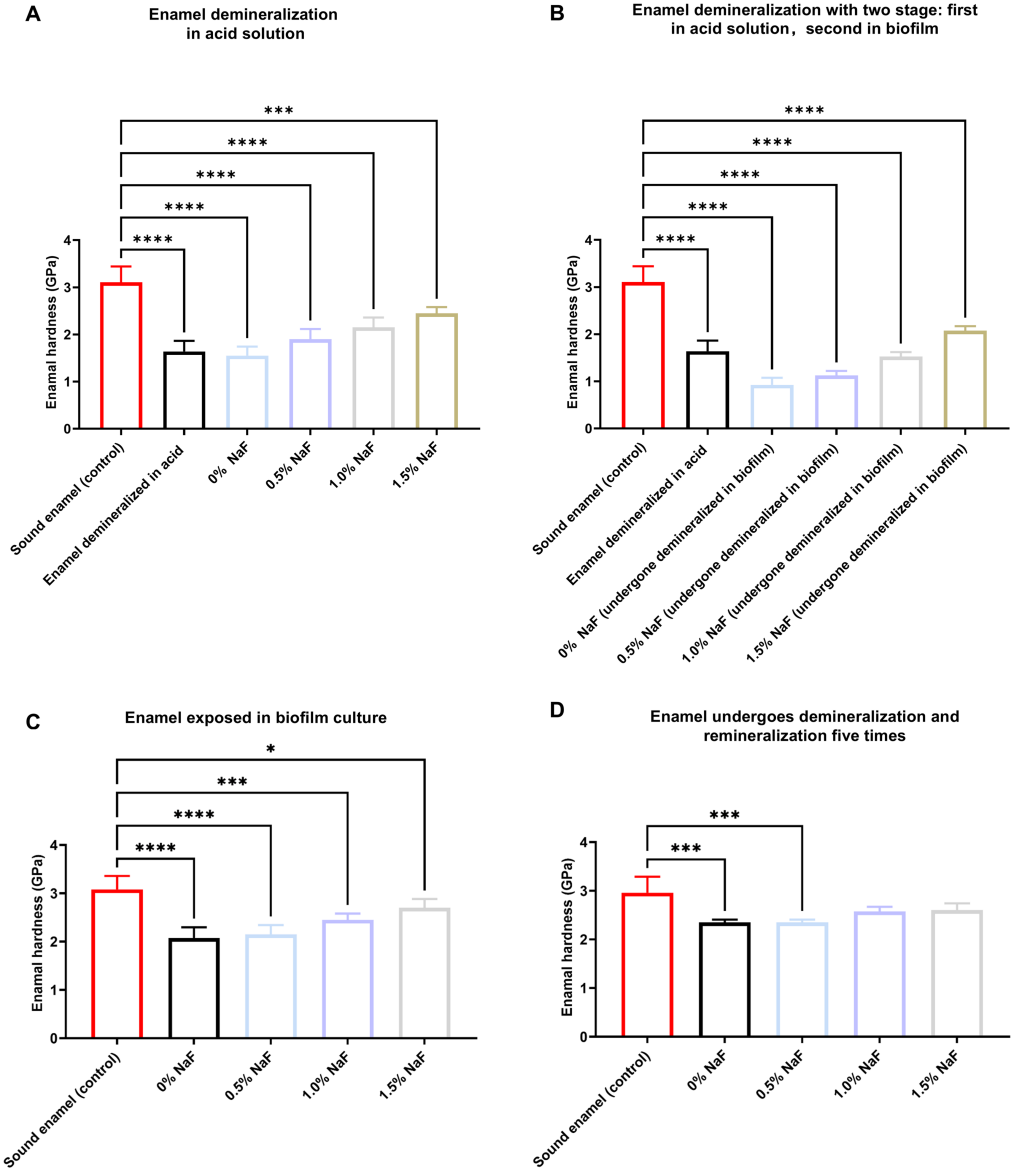
Enamel hardness assessment. **(A)** Enamel samples were demineralized in an acid solution. **(B)** Enamel samples were first demineralized like **(A)** and then demineralized under biofilm. **(C)** Untreated enamel samples under biofilm. **(D)** The enamel samples were demineralized as described in **(A)** and subsequently treated with a remineralizing solution that was reused five times. The results are presented as mean ± SD (*p < 0.05, ***p < 0.001 or ****p < 0.0001).

Enamel hardness decreased significantly after demineralization with an acid solution (*p* < 0.05), with recovery after incubation with the novel silicone appliance material (*p* > 0.05, Figure 6A). Following two stages of demineralization using acid solution and biofilm culture, enamel samples without NaF exhibited the lowest hardness values (*p* < 0.05) compared to samples with NaF. Hardness increased significantly with higher NaF concentrations in the silicone appliance material (*p* < 0.05, Figure 6B). Enamel demineralization was alleviated more effectively in a biofilm environment with increasing NaF concentrations (Figure 6C). After five sequential demineralization and remineralization treatment rounds, all NaF-containing samples exhibited significant improvements in enamel hardness compared to the NaF-free group. Notably, samples containing 1 and 1.5% NaF exhibited significantly higher hardness values than those with 0.5% NaF (Figure 6D).

## 4 Discussion

Incorporating anti-caries agents into orthodontic appliances to address WSLs and caries risk remains a significant challenge in current research. In this study, NaF was introduced into a removable silicone appliance for the first time, resulting in sustained and effective fluoride release and inhibiting cariogenic microorganisms. This approach significantly improved enamel resistance to demineralization and suppressed both the initiation and progression of dental caries. The novel material was hypothesized to maintain satisfactory mechanical properties without compromising biocompatibility. The comparable activity of murine L-929 fibroblasts, human gingival epithelial cells, and human oral keratinocytes between the NaF-infused material and the control group supports this hypothesis. These findings indicate that the NaF-infused appliance material significantly inhibits *S. mutans* activity, reduces acid production, and provides resistance enamel demineralization. Thus, this novel material provides a promising preventive strategy for WSLs and caries associated with orthodontic treatment in future clinical practice.

To assess the potential impact of NaF incorporation on the mechanical properties of silicone appliances and its subsequent effect on tooth movement, this study revealed that incorporating NaF at a maximum concentration of 1.5% did not significantly alter the Shore hardness, tear strength, tensile strength, flexural elastic modulus, tensile elastic modulus, and compression elastic modulus of the appliance, consistent with the findings of the control group (*p* > 0.05). Hence, this research maintained the NaF fusion concentration in the silicone appliance material at ≤1.5% for subsequent investigations.

Multiple factors, including temperature, humidity, oxygen levels, light exposure, material molding processes, and sterilization methods, influence the biocompatibility of orthodontic materials (Xu et al., 2021; Li et al., 2024). Cell viability is a critical indicator of the cytotoxic potential of tested materials, with higher viability percentages indicating lower cytotoxicity. Cell viability was assessed upon the incorporation of NaF, according to the ISO 10993 grading classification system, which categorizes materials with >80% cell viability as exhibiting slight cytotoxicity. These findings are consistent with previous studies evaluating the biocompatibility of calibration materials (Xu et al., 2021; Lambart et al., 2022; Li et al., 2015). In this study, at a NaF concentration of 1.5%, the average cell viability was >85%, indicating a classification between “non-cytotoxic” and “slightly cytotoxic.” Regarding other NaF concentrations, cell viability exceeded 90%.

Orthodontic appliances influence the composition of the oral microbiota primarily through plaque accumulation and disruption of oral hygiene (Santonocito and Polizzi, 2022). The extensive coverage of tooth surfaces by these appliances can induce quantitative and qualitative changes in the oral microbiota, which are critical factors during orthodontic treatment. WSLs, an early indicator of dental caries, are a common side effect associated with orthodontic appliances (Paula et al., 2017). WSLs develop by biofilm formation by cariogenic microorganisms, primarily *S. mutans*, which produce lactic acid, leading to enamel demineralization. Dental caries progression decreases the diversity of oral microorganisms, fostering the proliferation of cariogenic species and a gradual decline in pH. This imbalance between demineralization and remineralization results in mineral loss, ultimately leading to dental caries (Zou et al., 2018). Therefore, preventing biofilm formation and minimizing the colonization of cariogenic microorganisms are crucial for WSL prevention (Abdullah and John, 2016; Horst, 2018). This study demonstrated that incorporating NaF into the new material at concentrations ≥1.5% significantly inhibited *S. mutans* biofilm formation. The silicone appliance material with 1.5% NaF exhibited a marked inhibitory effect on both biofilm growth and acid production in salivary samples from children with S-ECC. Consequently, this novel NaF-infused silicone appliance material can prevent dental caries by regulating *S. mutans* biofilm activity and salivary-derived biofilms associated with S-ECC, promoting oral microbiota homeostasis.

According to previous studies, fluoride can inhibit surface demineralization and promote remineralization by supplying essential mineral ions (Ten Cate, 2004; Pitts et al., 2017). Additionally, research indicates fluoride affects oral bacteria, dental plaque, bacterial colonization, plaque formation, multi-species interactions, and various enzymatic activities (Oh et al., 2017; Ten Cate, 2004; Hamilton, 1990; Wiegand et al., 2007). The novel orthodontic material incorporated with NaF inhibited several physiological characteristics of *S. mutans* and saliva biofilms, suggesting that the material not only released mineral ions but also possessed antibacterial properties. Furthermore, it inhibited the development of saliva biofilms in multi-strain environments associated with S-ECC while promoting biofilm homeostasis. Upon fluoride release, the liberated fluoride ions penetrate bacterial cell membranes and disrupt metabolic activities, ultimately impairing bacterial growth and metabolism (Kaufmann and Bartholmes, 1992). The efficient fluoride-releasing material can modulate the balance of plaque microecology. However, the specific bacterial populations affected remain unclear, warranting further investigation.

The saliva-derived biofilms constructed *in vitro* closely resemble the plaque biofilm in the oral cavity, containing most microorganisms in dental plaque. This model simulates the oral microenvironment effectively while minimizing individual variability during *in vivo* experiments. Its advantages include good repeatability, ease of contamination, low cost, and high throughput (Keegan et al., 2012; Liang et al., 2020). In this research, salivary samples from children with S-ECC were collected to simulate the oral biofilm environment to evaluate the antibacterial effects of fluoride-containing silicone orthodontic materials in clinical practice.

Experiments on demineralization, remineralization, and multiple cycles simulating the oral microbial environment demonstrated that the novel orthodontic material effectively released fluoride. It also enhanced the hardness of bovine tooth enamel blocks subjected to demineralization, inhibiting the formation of WSLs during orthodontic treatment. Furthermore, the material facilitated enamel remineralization where WSLs had already formed, indicating significant anti-caries properties. Research suggests that fluoride also aids in sealing dentinal tubules, improving acid resistance, and promoting mineralization by serving as a source of fluoride ions (Matsuda et al., 2022). In this investigation, enamel hardness was primarily evaluated to assess enamel remineralization, and the study further explored whether the material could effectively occlude dentinal tubules, enhancing dentin’s resistance to acidic conditions.

Directly bonding silicone orthodontic materials is associated with challenges. On the other hand, periodontal and mucosal membranes might be damaged in animal models, possibly compromising the integrity of the epithelial binding, disrupting the balance of the periodontal and oral microenvironments, and affecting experimental accuracy. Therefore, this study used a more advanced model simulating S-ECC saliva biofilm in conjunction with bovine enamel demineralization and remineralization experiments to assess its anti-caries effects. However, it is important to note that the fluoride release cycle in this study was relatively short. Recharging with fluoride-coated modules should be considered to enhance the decay prevention capabilities of these appliance materials in clinical settings as part of future research efforts (Wei et al., 2024).

## 5 Conclusion

This study incorporated NaF into a silicone appliance to impart sustained antibacterial properties and prevent enamel demineralization. Therefore, the silicone appliance containing NaF has potential clinical applications in orthodontics. However, further research is necessary to improve the sustained release of fluoride. Further studies are needed due to the limited *in vivo* evidence before it can be translated into clinical practice.

## Data Availability

The raw data supporting the conclusions of this article will be made available by the authors, without undue reservation.
