# Supplementary material for: Anti-caries effect of a novel elastic silicone appliance material incorporating sodium fluoride
Source: Front Microbiol. 2025 Jan 6;15:1517188. doi: 10.3389/fmicb.2024.1517188 (PMC11743255; doi:10.3389/fmicb.2024.1517188)
Supplement: Supplementary file 1 [file Data_Sheet_1.docx]

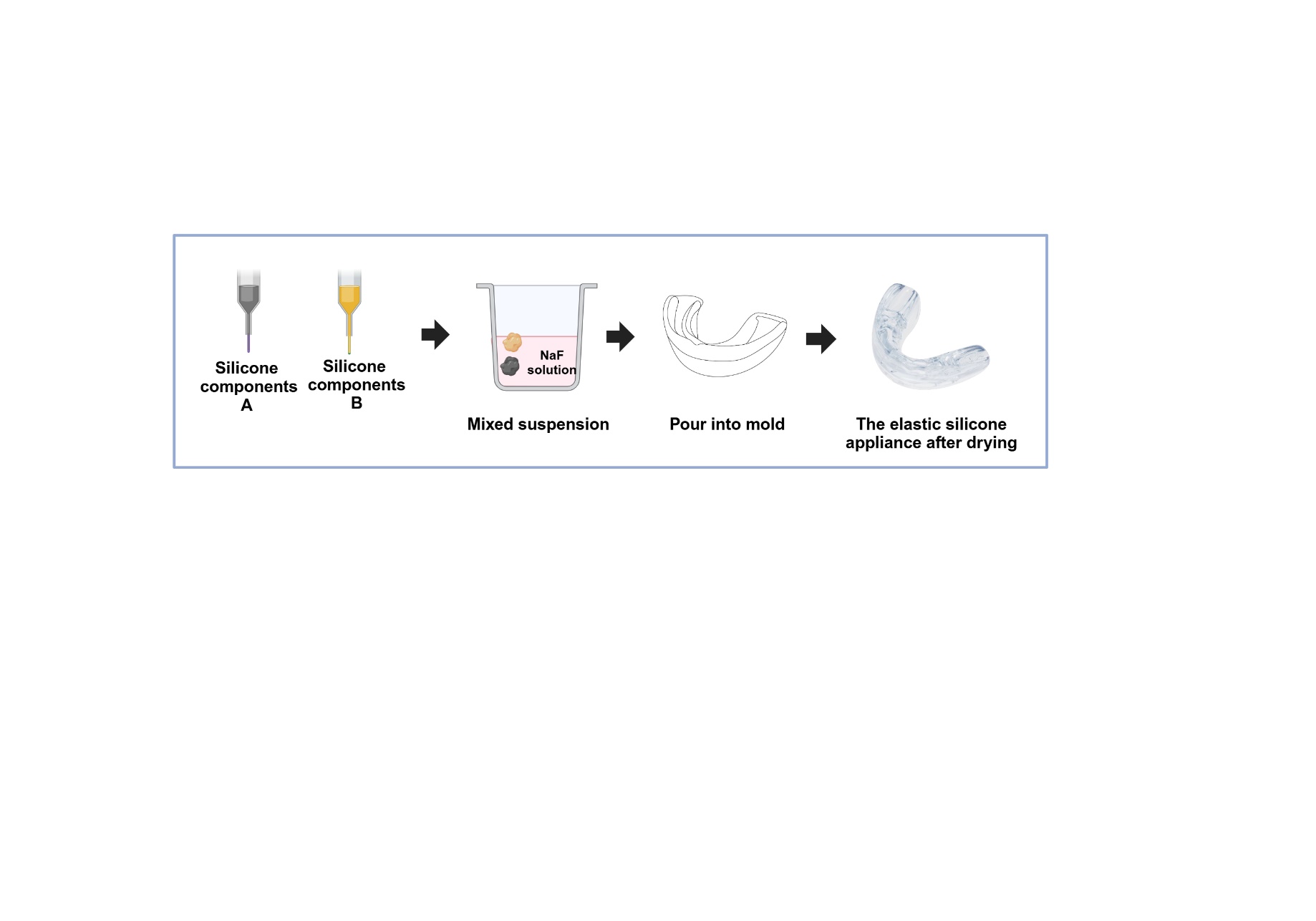


**Figure S1. The fabrication of a NaF-containing silicone orthodontic appliance.** The silicone components A and B are mixed together. Subsequently, a NaF solution is added to ensure uniform distribution throughout the mixture. The blend is then poured into a specialized mold and allowed to cure under specified conditions until solidified. After curing, the appliance is dried to remove any residual moisture and subsequently disinfected using methods appropriate for medical-grade silicone, ensuring its safety for patient use.


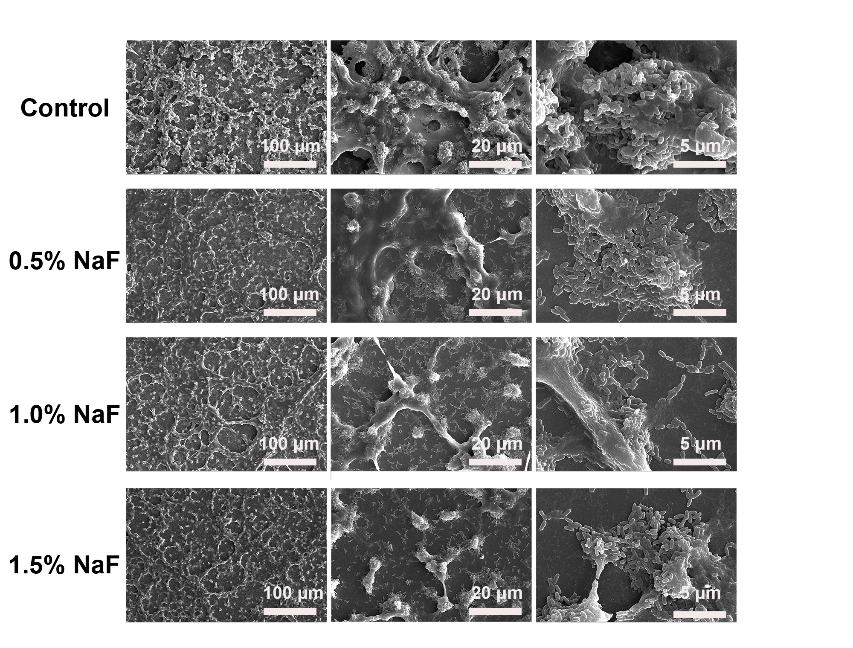


**Figure S2. Effect of silicone appliance material incorporating 0.5%, 1% and 1.5% NaF on biofilm formation *S. mutans* via scanning electron microscope (SEM).** SEM images showing the architecture of 24 hours *S. mutans* biofilms in different NaF concentrations. Images were taken at 1,000×, 5,000× and 20,000× magnification.
